# Successful pembrolizumab treatment in a patient with metastatic urothelial carcinoma and underlying overlap syndrome involving systemic sclerosis and systemic lupus erythematosus

**DOI:** 10.1002/iju5.12175

**Published:** 2020-06-16

**Authors:** Masayuki Kurokawa, Sei Naito, Suguru Ito, Satoshi Takai, Yuko Kawamura, Hisashi Kaneko, Hiroshi Kakizaki, Norihiko Tsuchiya

**Affiliations:** ^1^ Department of Urology Yamagata University Faculty of Medicine Yamagata City Japan; ^2^ Department of Urology Nihonkai General Hospital Sakata Yamagata Japan

**Keywords:** autoimmune disease, metastatic urothelial carcinoma, overlap syndrome, pembrolizumab, renal pelvic cancer

## Abstract

**Introduction:**

The safety and efficacy of pembrolizumab administration in patients with urothelial carcinoma and underlying autoimmune disease (including overlap syndrome) is unknown.

**Case presentation:**

We present the case of a 67‐year‐old woman with cT3N2M0 metastatic renal pelvic cancer who had been treated with prednisolone for overlap syndrome involving systemic sclerosis and systemic lupus erythematosus for 20 years. She had a remarkable response to pembrolizumab as a third‐line systemic therapy, wherein the tumor reduced in size and all regional lymph node and pulmonary metastases disappeared. She did not develop any immune‐related adverse events or autoimmune disease flare‐ups during the treatment.

**Conclusion:**

This case report suggests that pembrolizumab could be beneficial to patients with urothelial carcinoma and underlying well‐controlled overlap syndrome.

**Abbreviations & Acronyms**
AE: adverse event
AID: autoimmune diseases
CT: computed tomography
GC: gemcitabine plus cisplatin therapy
irAE: immune‐related adverse event
MVAC: methotrexate, vinblastine, adriamycin and cisplatin therapy
NSCLC: non‐small‐cell lung carcinoma
PD: programmed death
PD‐L1: programmed death‐ligand 1
SLE: systemic lupus erythematosus
SSc: systemic sclerosis

**Keynote message**

The safety and efficacy of using PD‐1 inhibitors in patients with underlying AID is unknown. We present the case of a 67‐year‐old woman with metastatic urothelial carcinoma and pre‐existing well‐controlled overlap syndrome involving SSc and SLE. This case report suggests that pembrolizumab could be beneficial to patients with urothelial carcinoma and underlying well‐controlled overlap syndrome.

## Case presentation

**Fig. 1:**
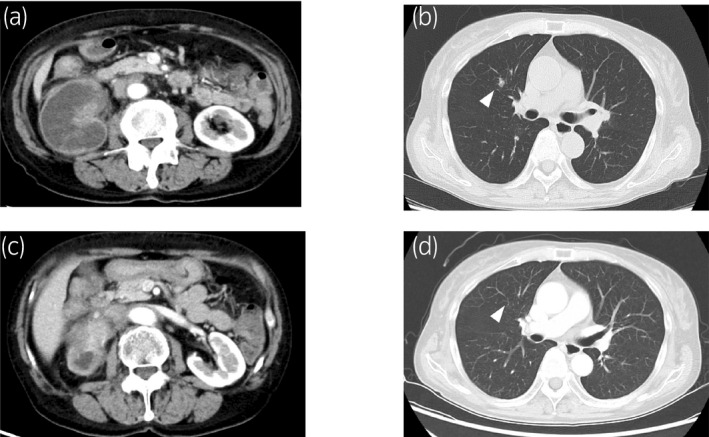
(a) CT scan shows a right renal pelvic mass and para‐aortic lymph node swelling at the time of pembrolizumab initiation. (b) CT scan shows a lung metastasis (arrowhead) at the time of pembrolizumab initiation. (c) CT scan shows a primary right renal pelvic tumor shrinkage after 6 months from pembrolizumab initiation. (d) CT scan shows disappearance of lung metastasis (arrowhead) after 6 months from pembrolizumab initiation.

A 67‐year‐old woman was admitted with suspicion of a right renal tumor at a general checkup ultrasound examination. CT showed a right renal tumor of 10 cm with invasion into the peripelvic fat and two para‐aortic lymph node metastases. Retrograde pyelography revealed a filling defect in the lower calyx but urine cytology was suspicious. To make a definite diagnosis, percutaneous renal tumor biopsy was performed under ultrasound guidance. Renal tumor pathology revealed urothelial carcinoma. Finally, she was diagnosed with cT3N2M0 renal pelvic carcinoma. She had been diagnosed with overlap syndrome involving SSc and SLE 20 years ago because of malar rash, photosensitivity, Raynaud’s phenomenon, swollen fingers, a high anti‐centromere antibody titer (index: 152.0, normal range <10), and a high anti‐nuclear antibody titer (1:640). She had been treated for overlap syndrome with a maintenance dose of prednisolone 2.5–5 mg; overlap syndrome had been well‐controlled and asymptomatic for several years. Induction systemic treatment was initiated with gemcitabine (1000 mg/m^2^), which was administered on days 1, 8, and 15, and cisplatin (70 mg/m^2^) on day 1 of a 28‐day cycle (GC). She experienced grade 3 neutropenia and thrombocytopenia during GC treatment. However, the para‐aortic lymph node metastases progressed after three cycles. Therefore, we continued the second‐line systemic therapy comprising methotrexate (30 mg/m^2^) on days 1 and 15, along with vinblastine (3 mg/m^2^), doxorubicin (30 mg/m^2^), and cisplatin (70 mg/m^2^) on day 2 for a 28‐day cycle (MVAC). She experienced grade 2 anorexia, grade 3 thrombocytopenia, and grade 4 neutropenia. After three cycles of MVAC therapy, she developed multiple lung metastases (Fig. 1a,b). Pembrolizumab was administered as a third‐line systemic therapy every 3 weeks. The primary tumor reduced in size and all lymph nodes and pulmonary metastases disappeared (Fig. 1c,d). The response continued until the latest follow‐up date, which was 6 months from the initiation of pembrolizumab. Daily administration of prednisolone (2.5 mg) was continued throughout pembrolizumab therapy, and she did not develop any AEs including irAEs or overlap syndrome flares. Anti‐nuclear antibody (1:640–1:1280) and centromere antibody titer (index: 142–152) did not change significantly during treatment and were associated with neither treatment response nor AEs.

## Discussion

Pembrolizumab is an immune‐oncologic drug that acts to inhibit PD‐1, a cell surface receptor that regulates T‐cell activation. It is recommended in the guidelines from the National Comprehensive Cancer Network^1^ and the European Urological Association^2^ for patients with platinum‐based chemotherapy‐resistant urothelial carcinoma. Clinical trials with PD‐1 inhibitors exclude patients with pre‐existing AID because of concerns associated with an increased risk for developing serious irAEs.^3^ In addition, corticosteroids are commonly used to treat AID, and they potentially have an immunosuppressive effect.^4^ Therefore, the safety and efficacy of PD‐1 inhibitors in such patients is unclear. Our patient had a remarkable response to pembrolizumab with no AEs.

SSc may overlap with SLE.^5^ It is important to regularly monitor serious SSc complications, such as pulmonary arterial hypertension, pulmonary fibrosis, malignant hypertension and acute renal failure.^5^ SSc‐SLE overlap syndrome patients are typically female, Asian, tend to be younger than pure SSc patients at diagnosis, and more frequently have pulmonary fibrosis than those with pure SSc.^6^ Throughout the course of pembrolizumab treatment, our case did not develop any skin lesions, renal crises, or interstitial lung disease.

Some retrospective studies have reported that immune‐oncologic treatment for patients with underlying AID is generally manageable. In a study comprising 52 melanoma patients with pre‐existing AID treated with PD‐1 inhibitor, 38% of patients had flare‐ups and 20% developed irAEs, of which 10% were grade 3 with no grade 4 or 5 events.^7^ In another study comprising 56 patients with AID and NSCLC, who were treated with PD‐1/PD‐L1 inhibitor, 23% developed flare‐ups and 38% developed irAEs, of which 26% were grade 3 or 4 with no grade 5 events.^8^ These rates of AEs were similar to the trials, wherein patients with AID were excluded.^7^, ^8^ In addition, 50% of the patients with NSCLC and symptomatic AID at the time of PD‐1/PD‐L1 inhibitor initiation developed flare‐ups. On the other hand, only 18% of the other patients who were initially asymptomatic developed flare‐ups.^8^ In our case, the overlap syndrome had been well‐controlled for several years and the patient was asymptomatic at the time of pembrolizumab initiation. Thus, the possibility of a flare‐up was considered to be low.

Corticosteroids are commonly used in patients with AID. Those drugs suppress the immune system by inhibiting lymphocyte activation and inducing apoptosis in lymphocytes.^9^ The immunosuppressive effect of corticosteroids can theoretically reduce the efficacy of PD‐1 inhibitors. In a retrospective study of patients with NSCLC treated with PD‐1/PD‐L1 inhibitors, baseline corticosteroid use of more than 10 mg of prednisone‐equivalent was correlated with worse clinical outcome compared with those who administrated <10 mg of prednisone‐equivalent.^10^ Generally, stable SLE or SSc patients are maintained on low‐dose glucocorticoids (2.5–7.5 mg/day).^11^, ^12^ Three cases with SSc or SLE treated with PD‐1 inhibitors were reported in the NSCLC study, but detailed information on the treatments and efficacy was not described in the report.^8^ In an NSCLC and SLE case treated with pembrolizumab, who had been well controlled with prednisolone 5 mg and tacrolimus 1 mg, complete remission was attained.^13^ Our patient also had been treated with 2.5–5 mg prednisolone for 20 years and we speculate that those low‐dose corticosteroid (in the range of physiologic adrenal secretion) might not affect the response to pembrolizumab.

Further studies are required to clarify the safety and efficacy of pembrolizumab and optical maintenance dose of immunosuppressants in patients with AID.

## Conclusions

This case report suggests that pembrolizumab could be beneficial to patients with urothelial carcinoma and pre‐existing well‐controlled AID.

## Conflict of interest

The authors declare no conflict of interest.
